# LncRNA plasmacytoma variant translocation 1 is an oncogene in bladder urothelial carcinoma

**DOI:** 10.18632/oncotarget.19604

**Published:** 2017-07-26

**Authors:** Zhongyuan Liu, Hui Zhang

**Affiliations:** ^1^ Department of Urinary Surgery, Shengjing Hospital, China Medical University, Shenyang 110004, China

**Keywords:** bladder cancer, long noncoding RNA, plasmacytoma variant translocation 1, chemoresistance, Wnt/β-catenin pathway

## Abstract

Bladder cancer (BC) is the most lethal malignant cancer of the genitourinary system, and bladder urothelial carcinoma (BUC) is the most common type of BC. The long non-coding RNA (lncRNA) plasmacytoma variant translocation 1 (PVT1) is overexpressed in several malignant tumors, including BC. Using a lncRNA array and quantitative real-time PCR, we detected greater expression of PVT1 in BUC tissues and cell lines resistant to doxorubicin (DOX) and cisplatin (DDP) than in DOX- and DDP-sensitive cells. PVT1 knockdown reduced proliferation and invasion by a DOX- and DDP-resistant T24/DR BUC cells, arrested cells in G1 phase, and increased apoptosis. PVT1 knockdown also sensitized T24/DR cells to DOX and DDP, and suppressed expression of multidrug resistance 1 (MDR1) and multidrug resistance associated protein 1 (MRP1). Wnt/β-catenin pathway activation in T24/DR cells reversed the effects of PVT1 knockdown on metastasis-associated behavior and chemoresistance. In sum, lncRNA PVT1 is overexpressed in multidrug resistant BUC tissues and cell lines, and PVT1 knockdown reduces BUC cell proliferation, invasiveness, and chemoresistance by modulating Wnt/β-catenin signaling. These results provide new insight into BUC chemoresistance mechanisms and suggest potential therapeutic targets for anti-BUC therapeutics.

## INTRODUCTION

Bladder cancer (BC) is the most lethal malignant cancer of the genitourinary system, with bladder urothelial carcinomas (BUCs) the most common type of BC [1]. Anti-BUC therapeutic strategies include surgical resection, systemic chemotherapy, and intravesical chemotherapy [2]. However, even with recent improvements in therapeutic options, the prognosis of BUC patients is still poor [3]. Adjuvant chemotherapy may decrease BUC cell metastasis and improve patient survival, but resistance to chemotherapeutics has severely limited the efficacies of these drugs in clinical BUC applications. Elucidating the mechanisms underlying BUC chemoresistance and identifying novel therapeutic targets will be crucial to further improvements in BUC patient prognosis.

Long noncoding RNA (lncRNA) dysregulation is associated with aberrant biological processes promoting a variety of diseases, including cancers [4–6]. With the development of bioinformatics and functional genomics tools, abnormal lncRNA expression was discovered in some malignant tumors. These lncRNAs were related to tumorigenesis and disease progression, appearing to function as oncogenes and tumor suppressors [7, 8].

The plasmacytoma variant translocation 1 (PVT1) lncRNA maps to 8q24.21. Consistent with its association with cancer, *PVT1* transcription is regulated by the tumor suppressor, TP53, through a canonical TP53-binding site, and PVT1 may regulate the proto-oncogene, MYC, to promote tumorigenesis [9, 10]. PVT1 is a candidate oncogene in many tumor types, including lung, gastric, and prostate cancers [11–13]. Zhuang, *et al*. found that PVT1 was upregulated in BC, where it promoted tumor cell proliferation and suppressed apoptosis [14]. However, the effects of PVT1 on chemoresistance in BUC remain poorly understood.

## RESULTS

### PVT1 overexpression correlated with BUC cell chemoresistance

**Figure 1:**
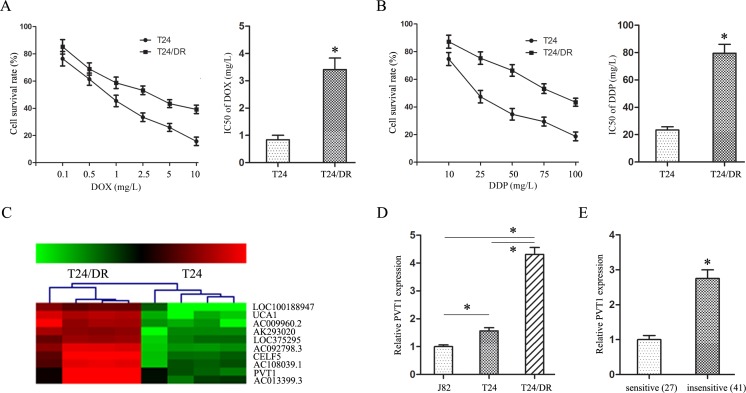
PVT1 overexpression correlated with DOX and DDP resistance in BUC cells DOX **(A)** and DDP **(B)** dose-response curves and IC50s in T24 and T24/DR cells. lncRNA microarray analysis of total RNA isolated from T24 and T24/DR cells **(C)** 1–4: T24/DR cells; 1N–4N: T24 cells. PVT1 expression in J82, T24, and T24/DR cells **(D)** PVT1 expression in BUC patient tissues treated with DOX and DDP **(E)** *P<0.05.

The half maximal inhibitory concentrations (IC50s) of doxorubicin (DOX) and cisplatin (DDP) in T24/DR cells were 3.41±0.42 mg/L and 79.62±6.42 mg/L, respectively, and in T24 cells were 0.84±0.16 mg/L and 23.47±2.33 mg/L, respectively (*P*<0.05; Figure 1A–1B). T24/DR cells were more resistant to DOX and DDP than T24 cells; their respective resistance indices (RI) were 4.06 and 3.39.

To identify lncRNAs associated with BUC chemoresistance, the chemoresistant (T24/DR) and control cells (T24) were examined using a lncRNA array. PVT1 was upregulated over eight-fold in T24/DR cells (Figure 1C), and this overexpression was confirmed via qRT-PCR assay (Figure 1D).

*PVT1* expression in BUC patients treated with DOX and DDP was examined using qRT-PCR. PVT1 expression in the insensitive group was much higher than in the sensitive group (Figure 1E). This result revealed that *PVT1* expression was negatively correlated with BUC patient response to DOX and DDP, suggesting that PVT1 promotes chemoresistance in BUC.

### PVT1 knockdown inhibited T24/DR cell malignancy-associated behaviors

**Figure 2:**
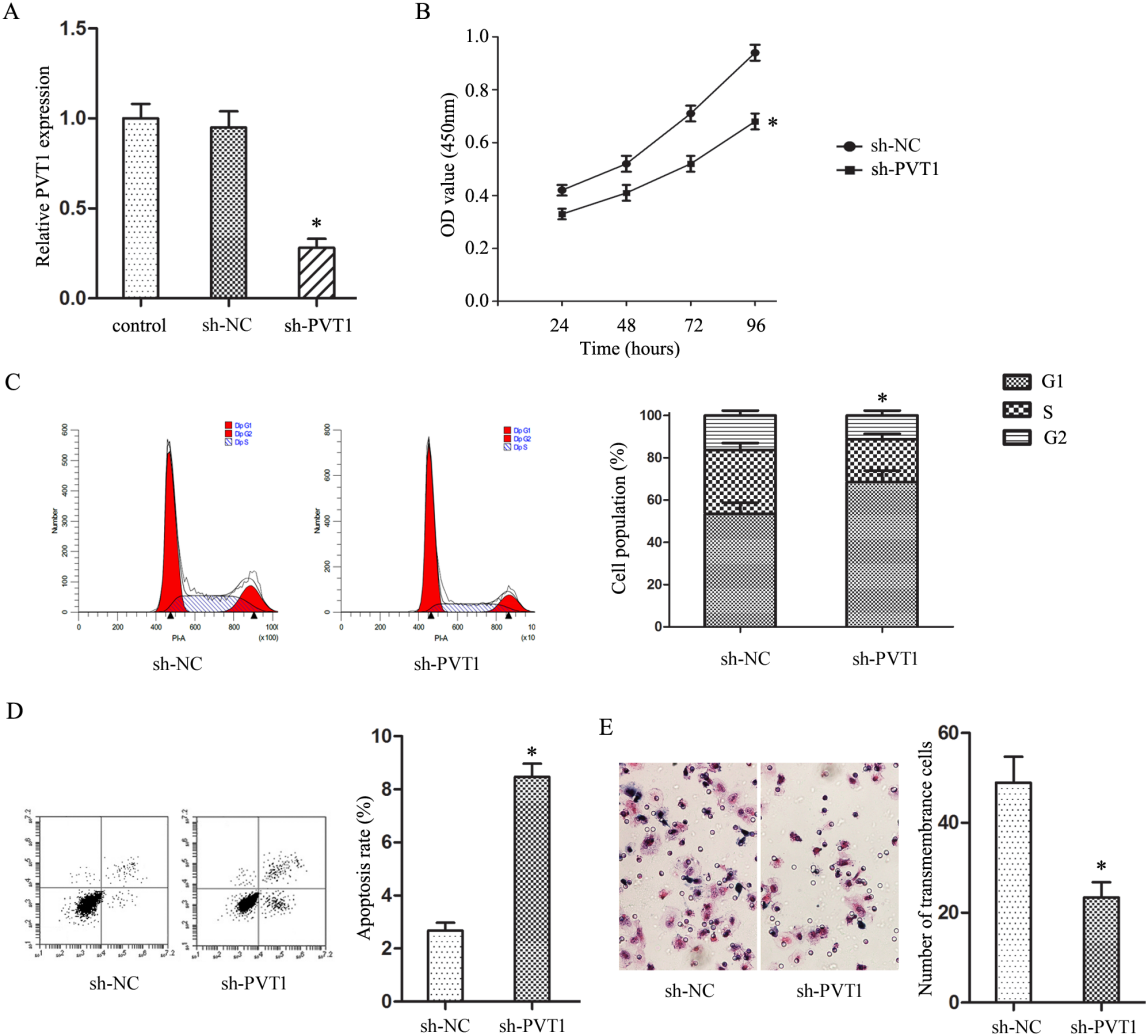
PVT1 knockdown inhibited T24/DR cell malignancy-associated characteristics PVT1 expression in T24/DR cells after sh-PVT1 transfection **(A)** T24/DR cell viability as detected by CCK8 assay **(B)** T24/DR cell cycle statuses **(C)** and apoptosis **(D)** as detected by flow cytometry. T24/DR cell invasion as measured via Transwell assay **(E)** *P<0.05 vs NC.

PVT1 was silenced in T24/DR cells via transfection with sh-PVT1 (Figure 2A). CCK8 assay results showed that PVT1 knockdown reduced T24/DR cell viability (*P*<0.05; Figure 2B), and arrested cells in G1 phase (*P*<0.05; Figure 2C). These results suggest that PVT1 participates in T24/DR cell proliferation. Additionally, flow cytometry analyses found that PVT1 knockdown increased T24/DR cell apoptosis (*P*<0.05; Figure 2D). Finally, Transwell assays showed that PVT1 knockdown reduced T24/DR cell invasion compared to controls (*P*<0.05; Figure 2E). Together, these data demonstrated that PVT1 knockdown inhibited T24/DR cell malignant behaviors.

### PVT1 knockdown sensitized T24/DR cells to DOX and DDP

**Figure 3:**
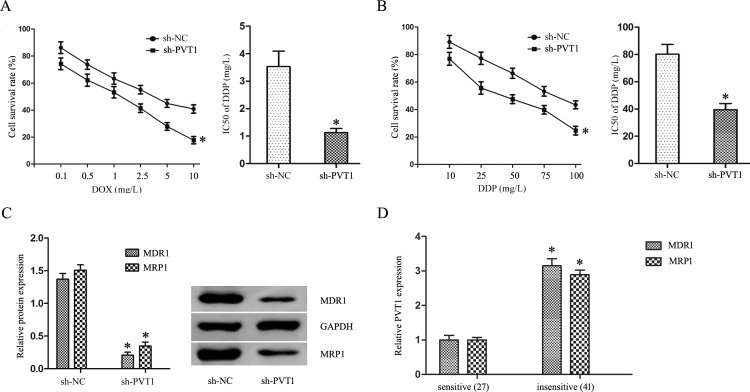
PVT1 knockdown sensitized T24/DR cells to DOX and DDP DOX **(A)** and DDP **(B)** dose-response curves and IC50s in T24 and T24/DR cells. MDR1 and MRP1 expression in T24/DR cells **(C)** and in BUC patients treated with DOX and DDP **(D)** *P<0.05 vs NC.

DOX and DDP combination chemotherapy is a first-line treatment for BUC patients. DOX and DDP IC50s in T24/DR cells were reduced following PVT1 knockdown, from 3.53±0.57 mg/L and 80.31±7.15 mg/L to 1.13±0.15 mg/L and 39.52±4.48 mg/L, respectively (*P*<0.05; Figure 3A–3B). Western blotting also showed that PVT1 knockdown inhibited multidrug resistance protein 1 (MDR1) and multidrug resistance associated protein 1 (MRP1) expression in T24/DR cells (*P*<0.05; Figure 3C). MDR1 and MRP1 were upregulated in insensitive BUC patients compared to sensitive patients (*P*<0.05; Figure 3D). Pearson's correlation analysis showed that MDR1 and MRP1 levels were positively correlated with PVT1 expression in BUC patients (r=0.292, P=0.041 and *r*=0.352, P=0.001, respectively).

### PVT1 knockdown inhibited Wnt/β-catenin signaling

Gene Ontology (GO) analysis of lncRNAs differentially expressed between T24/DR and T24 cells showed that cell cycle and cell adhesion were the major enriched processes. Wnt/β-catenin signaling was the most enriched pathway according to Kyoto Encyclopedia of Genes and Genomes (KEGG) pathway analysis. To assess potential correlations between PVT1 and Wnt/β-catenin signaling in BUC cells, we examined the impact of PVT1 knockdown on Wnt/β-catenin activity.

**Figure 4:**
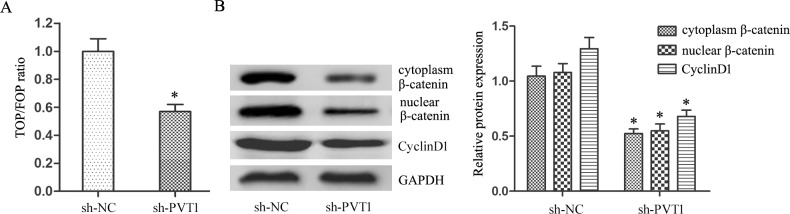
PVT1 knockdown inhibited Wnt/β-catenin signaling in T24/DR cells Ratio of TOP/FOP values in T24/DR cells as measured via luciferase assay **(A)** cytoplasmic and nuclear β-catenin and CyclinD1 expression in T24/DR cells **(B)** *P<0.05.

TOP/FOPflash luciferase reporter assay results showed that PVT1 knockdown suppressed the TOP/FOP ratio in T24/DR cells (*P*<0.05; Figure 4A). PVT1 knockdown also reduced both cytoplasmic and nuclear β-catenin levels (*P*<0.05; Figure 4B) and CyclinD1 expression, the target gene of β-catenin (*P*<0.05; Figure 4B). These results suggest that PVT1 knockdown reduced Wnt/β-catenin signaling.

### Wnt/β-catenin pathway activation reversed the effects of PVT1 knockdown in T24/DR cells

**Figure 5:**
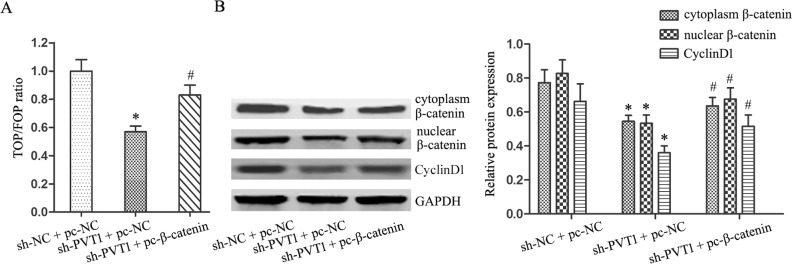
β-catenin overexpression reversed the inhibitory effects of PVT1 knockdown on Wnt/β-catenin signaling in T24/DR cells Ratio of TOP/FOP values in T24/DR cells as measured via luciferase assay **(A)** cytoplasmic and nuclear β-catenin and CyclinD1 expression in T24/DR cells **(B)** *P<0.05 vs sh-NC + pc-NC; #*P*<0.05 vs sh-PVT1 + pc-NC.

**Figure 6:**
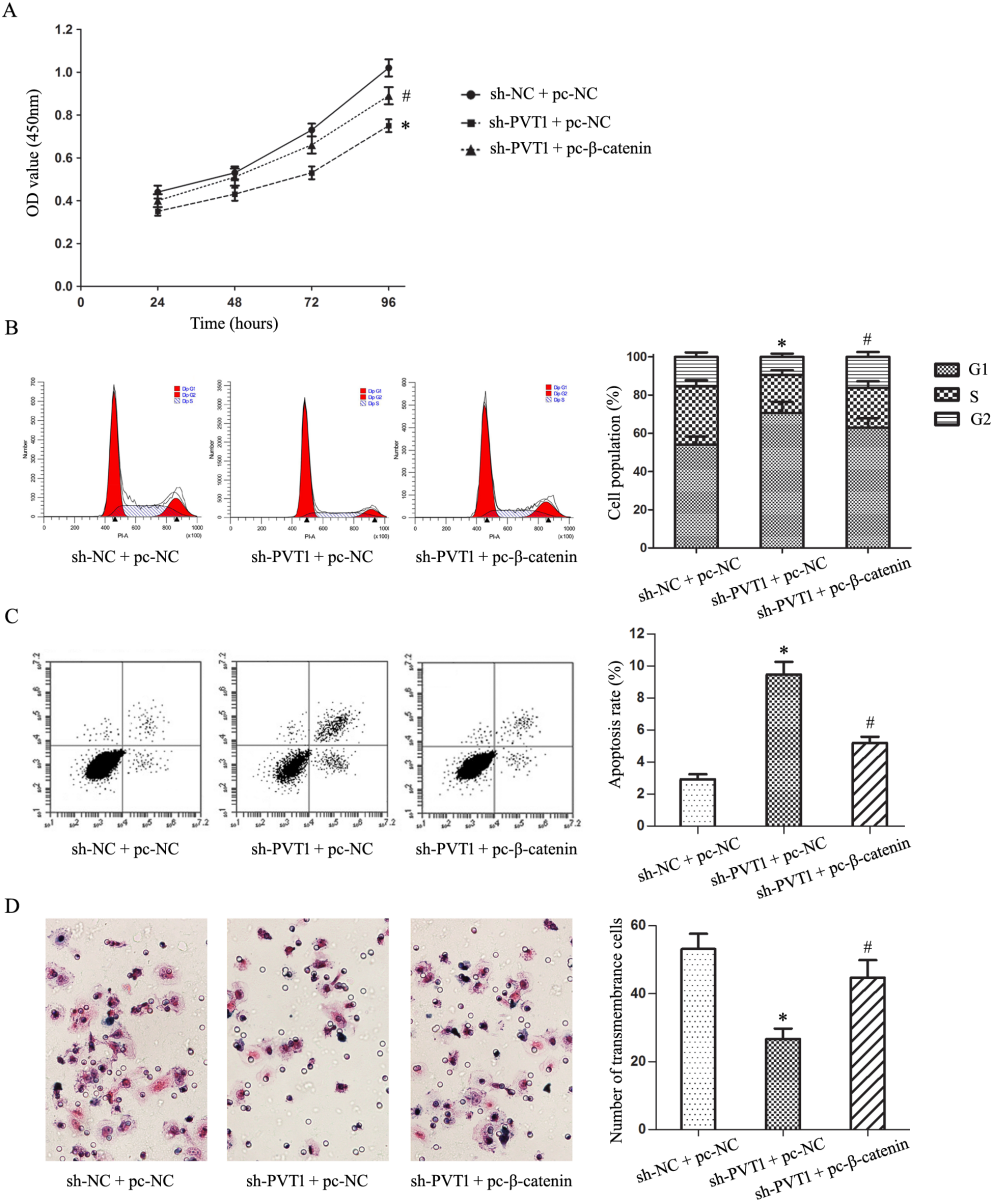
Wnt/β-catenin pathway-mediated inhibition of T24/DR cell malignant characteristics induced by PVT1 knockdown T24/DR cell viability as detected by CCK8 assay **(A)** T24/DR cell cycle statuses **(B)** and apoptosis **(C)** as detected by flow cytometry T24/DR cell invasion as measured via Transwell assay **(D)** *P<0.05 vs sh-NC + pc-NC; #*P*<0.05 vs sh-PVT1 + pc-NC.

**Figure 7:**
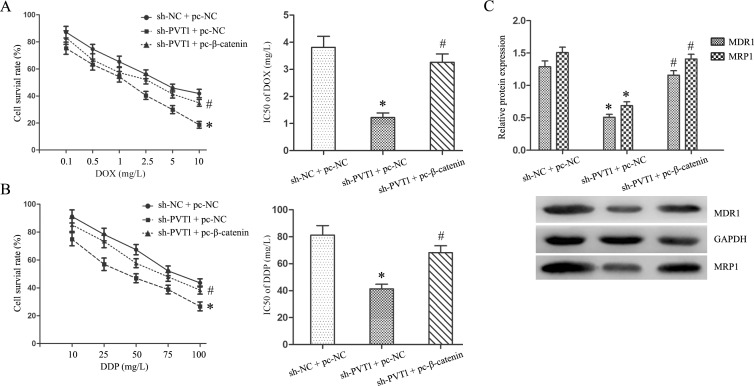
Wnt/β-catenin pathway activation restored DOX and DDP resistance in T24/DR cells sensitized by PVT1 knockdown DOX **(A)** and DPP **(B)** dose-response curves and IC50s in T24 and T24/DR cells. MDR1 and MRP1 expression in T24/DR cells **(C)** *P<0.05 vs sh-NC + pc-NC; #*P*<0.05 vs sh-PVT1 + pc-NC.

We transfected the β-catenin expression plasmid (pc-β-catenin) into T24/DR cells to activate Wnt/β-catenin signaling inhibited by PVT1 knockdown. TOP/FOP flash luciferase reporter assays and western blotting showed that β-catenin upregulation activated Wnt/β-catenin signaling (*P*<0.05; Figure 5A–5B). Wnt/β-catenin pathway activation reversed the effects of PVT1 knockdown on T24/DR cells, upregulating cell proliferation and invasion, and suppressing apoptosis (*P*<0.05; Figure 6A–6D). Wnt/β-catenin pathway activation also largely restored T24/DR cell chemoresistance reduced by PVT1 knockdown. DOX and DDP IC50s in T24/DR cells increased from 1.22±0.19 mg/L and 41.34±3.47 mg/L to 3.26±0.46 mg/L and 68.18±5.11 mg/L, respectively (*P*<0.05; Figure 7A–7B). MDR1 and MRP1 were also upregulated by Wnt/β-catenin pathway activation (*P*<0.05; Figure 7C–7D).

## DISCUSSION

Altered lncRNA expression contributes to human tumor development and progression, providing novel anti-tumor therapeutic target opportunities [15–17]. Recent studies reported lncRNA involvement in BUC cell chemoresistance. lncRNA HOTAIR promoted cell proliferation and inhibited chemosensitivity to DOX in bladder transitional cell carcinoma [18], and lncRNA CCAT1 promoted lung adenocarcinoma cell chemoresistance to docetaxel by sponging let-7c [19]. Most relevant studies have focused on chemoresistance to a single drug. Because combination chemotherapy is preferred in clinical BUC treatment, we established the T24/DR cell line, which is resistant to both DOX and DDP.

Chemotherapy is an effective approach to prevent BUC metastasis and recrudescence. However, chemoresistance is very common and mainly responsible for clinical treatment failures, leading to poor BUC patient prognosis. This study used a lncRNA array to identify lncRNAs associated with chemoresistance in BUC. lncRNA PVT1 was upregulated over eight-fold in T24/DR cells compared to parent (non-resistant) T24 cells. *PVT1* is aberrantly expressed in many tumor types, and was reported as a candidate oncogene [21, 22]. Zhuang, *et al*. found that *PVT1* was upregulated in BC, and was correlated with tumor histological grade and TNM stage [14]. PVT1 silencing inhibited BC cell growth and induced apoptosis [14], but the role of PVT1 in BUC chemoresistance was not assessed. Our study confirmed PVT1 overexpression in BUC chemoresistant tissues and cell lines, and revealed that PVT1 levels were negatively correlated with BUC patient response to DOX and DDP. Similarly, we found that PVT1 knockdown inhibited T24/DR cell proliferation and invasion, and enhanced apoptosis, suggesting that PVT1 acts as a potential oncogene in BUC. PVT1 knockdown also reduced T24/DR resistance to DOX and DDP, and downregulated MDR1 and MRP1 expression. MDR1 is an important ATP-dependent membrane protein that pumps many foreign substances out of cells [23]. MRP1 is a member of the ATP-binding cassette transporters superfamily, which transports various molecules across extra- and intra-cellular membranes [24]. Both MDR1 and MRP1 promote drug resistance by transporting anticancer drugs out of target cells before they reach the cytosol [25, 26]. We speculated that PVT1 might promote chemoresistance by regulating MDR1 and MRP1 expression. However, verification of this possible regulatory relationship requires further research.

GO analysis of lncRNA microarray data showed that cell cycle and cell adhesion were the most common processes associated with lncRNAs differentially expressed between T24/DR and T24 cells. Wnt/β-catenin signaling was the most enriched pathway according to KEGG analysis. Wnt/β-catenin signaling plays important roles in cancer by affecting tumor cell growth, development, and metabolism, and stem cell maintenance, and β-catenin is a key downstream effector [27–31]. We confirmed that PVT1 knockdown inhibited Wnt/β-catenin signaling via the TOP/FOPflash luciferase reporter system and western blotting. Zhang, *et al*. showed that PVT1 epigenetically silenced miR-200b expression to promote cervical cancer cell proliferation, migration, and cell cycle progression [32]. miR-200b inhibited Wnt/β-catenin pathway activation by silencing PCNA-associated factor (PAF) in esophageal squamous cell carcinoma [33]. We speculate that PVT1 knockdown inhibited Wnt/β-catenin signaling in our study through the miR-200b/PAF axis. Fan, *et al*. reported that UCA1 increased BC cell chemoresistance by regulating Wnt/β-catenin signaling [34]. Thus, we hypothesized that PVT1 might regulate BUC cell chemoresistance through Wnt/β-catenin signaling. We found that activating Wnt/β-catenin signaling by upregulating β-catenin reversed the effects of PVT1 knockdown in T24/DR cells, restoring chemoresistance in these cells.

In conclusion, lncRNA PVT1 was overexpressesed in chemoresistant BUC tissues and cell lines, and acted as a potential oncogene by promoting BUC cell chemoresistance and malignant behaviors through Wnt/β-catenin signaling. Our results provide new insights into BUC chemoresistance mechanisms, and suggest novel potential therapeutic targets for anti-BUC therapeutics.

## MATERIALS AND METHODS

### Cell culture

Human BUC J82 and T24 cell lines were obtained from the China Academy of Chinese Medical Sciences (Shanghai, China). Cells were cultured Dulbecco's Modified Eagle's Medium (DMEM) supplemented with 100 U/ml penicillin, 100 μg/ml streptomycin sulfates, and 10% fetal bovine serum (FBS; Gibco, Carlsbad, CA, USA), and maintained at 37°C in a humidified incubator with 5% CO_2_. The DOX- and DDP-resistant T24/DR cell line was established in our laboratory previously via co-culture with continuously increasing DOX and DDP (Sigma, St. Louis, MO, USA) concentrations [35].

### lncRNA microarray and functional enrichment analysis

Total RNA was extracted using the TRIzol reagent (Invitrogen, Foster City, CA, USA) according to the manufacturer's instructions. RNA was cleaned using the RNasey Mini Kit (p/n 74104, Qiagen, Germany), and labeled with Quick Amp Labeling Kit One-Color (p/n 5190-0442, Agilent, USA). lncRNA expression was detected using the lncRNA microarray (Arraystar, Rockville, MD, USA). Microarray data were extracted using Agilent Feature Extraction Software.

During functional enrichment analysis, the online software Database for Annotation, Visualization, and Integrated Discovery (DAVID; https://david.ncifcrf.gov/) was utilized to perform GO and KEGG pathway analyses to investigate potential pathways and networks associated with differentially expressed genes [36, 37]. Terms with *P*<0.05 were considered significantly enriched.

### Clinical specimens

68 BUC specimens were gathered from the Department of Urinary Surgery of Shengjing Hospital (Shenyang, Liaoning, China) from Mar 2014 to Sep 2015 through cystoscope. All BUC patients were treated with DOX and DDP, and chemotherapy effects were evaluated in accordance with RECIST Response Evaluation Criteria. Patients were divided into two groups; patients in the sensitive group (n=27) were relieved completely or partially, and patients in the insensitive group (n=41) were stable or deteriorating. This study was approved the Ethics Committees of Shengjing Hospital, and patient consent was achieved before surgery.

### Quantitative real time PCR (qRT-PCR)

Total RNA was extracted from BUC tissues and cells using the TRIzol reagent (Invitrogen, Foster City, CA, USA), and the One Step SYBR^®^ Green Master Mix Kit (QIAGEN, Hilden, GER) was used to detect *PVT1*, *MDR1*, and *MRP1* expression using the 7500 Real-Time PCR System (Applied Biosystems, USA) according to the manufacturer's instructions. Primers are listed in Table 1. Cycling conditions were as follows: Stage 1 (reverse transcription reaction): 42°C for 5 min, 95°C for 10 sec, 1 Cycle; Stage 2 (PCR): 95°C for 5 sec, 60°C for 34 sec, 40 Cycles; Stage 3 (dissociation curve analysis): 95°C for 15 sec, 65°C for 1 min, 95°C for 14 sec. *PVT1*, *MDR1*, and *MRP1* relative expression was normalized to GAPDH.

**Table 1:** The sequence for primers

Primers used for qRT-PCR	
PVT1 F	GCCCCTTCTATGGGAATCACTA
PVT1 R	GGGGCAGAGATGAAATCGTAAT
GAPDH F	CGCTCTCTGCTCCTCCTGTTC
GAPDH R	ATCCGTTGACTCCGACCTTCAC
MDR1 F	GGAGCCTACTTGGTGGCACATAA
MDR1 R	TGGCATAGTCA GGAGCAAATGAAC
MRP1 F	ATTCAGATGACACCTCTCAACAA
MRP1 R	TCCTTCTTCCAGTTCTTTACCAA

### Plasmid construction and cell transfection

The *PVT1* target interference sequence, CAGCCATCATGATGGTACT, was designed using Ambion's online siRNA Target Finder. Double stranded siRNA oligonucleotides were synthesized and inserted into the pSilencer 4.1 plasmid (Invitrogen, Carlsbad, CA, USA), which contained a neomycin resistance marker for selection of stable transfectants in the presence of G418. The recombinant plasmid, sh-PVT1, was constructed by the Genscript Corporation (Nanjing, China). The negative control plasmid, pSilencer-NC (sh-NC), encodes an siRNA with no significant sequence similarity to human genes. The full-length β-catenin sequence was synthesized and cloned into the expression plasmid, pcDNA-3.1 (Invitrogen, Carlsbad, CA, USA) between the HindIII and BamHI to construct the pc-β-catenin plasmid. Empty pcDNA-3.1 was used as a negative control (pc-NC). All plasmids were verified by DNA sequencing (Sangon Biotech, Shanghai, China). Plasmids were transfected into cells using Lipofectamine 3000 (Invitrogen, Carlsbad, CA, USA) according to the manufacturer's instructions. G418 (Sigma-Aldrich, St. Louis, MO, USA) was used to establish stably transfected cell lines.

### Cell proliferation and chemoresistance assay

For the cell proliferation assay, 2×10^4^ cells/well were seeded into 96-well plates. 10 μl Cell Counting Kit-8 reagent (CCK8; Beyotime, Jiangsu, China) was added to each well and incubated for 2 h at 37°C. Absorbance at 450 nm was measured using the SpectraMax M5 microplate reader (Molecular Devices, Sunnyvale, CA, USA).

For the chemoresistance assay, 3000 cells/well were seeded into 96-well plates and treated with DOX (0.1, 0.5, 1, 2.5, 5, or 10 mg/L) or DDP (10, 25, 50, 75, or 100 mg/L) 24 h later [34, 38]. After 48 h, cell viability was assessed, and dose-response curves and IC50 values were calculated using Graphpad5 software.

### Flow cytometry

For the cell cycle assay, 1×10^6^ cells were collected and washed with ice-cold PBS. Cells were resuspended in 10 ml of 70% ethanol for fixation overnight at −20°C. Cells were then washed twice with PBS, exposed to 500 μl RNase Diluent (Sigma-Aldrich, St. Louis, MO, USA), incubated at room temperature for 30 min, and then stained with propidium iodide (PI) (25 μl at 1 mg/ml; Sigma-Aldrich, St. Louis, MO, USA) for 5 min in the dark. Cell cycle status was determined using flow cytometry (FACScan; Becton Dickinson, Franklin Lakes, NJ, USA).

Apoptosis was detected using the Annexin V-FITC apoptosis detection kit (Biosea, Beijing, China) via flow cytometry. Briefly, after washing with cold PBS, 1×10^6^ cells/ml were resuspended in 1× binding buffer for 10 min. 100 μl cell suspension was incubated with 2.5 μl FITC-Annexin V and 2.5 μl PI for 10 min in the dark at room temperature. The reaction was terminated with the addition of 400 μl 1× binding buffer and analyzed via flow cytometry. Data were analyzed using Diva 8.0 software (Becton Dickinson, Franklin Lakes, NJ, USA). Cells in the right lower quadrant (FITC-Annexin V-positive and PI-negative) were regarded as early stage apoptosis.

### Transwell invasion assay

Cell invasion was assayed using Transwell chambers (Costar, Corning, NY, USA) coated with Matrigel (Becton Dickinson, Franklin Lakes, NJ, USA) according to the manufacturer's instructions. Briefly, 2×10^5^ cells were seeded onto upper chambers in 25 μl serum-free medium. Lower chambers were filed with 0.5 ml human NIH3T3 cell supernatant. After incubation for the appropriate time at 37°C with 5% CO_2_, cells invading to the lower surface were stained with haematoxylin & eosin (H&E), and photographed under a light microscope. Numbers of invading cells were counted in five random fields for each chamber.

### Western blotting

Cells were lysed using RIPA buffer (Beyotime, Shangahi, China) and total protein concentration was determined using the BCA assay (Beyotime, Shangahi, China). 30 μg of protein was subjected to 10% SDS-PAGE and transferred to a 0.22 μm PVDF membrane. The membrane was hybridized with rabbit β-catenin antibody (ab32572, Abcam, USA), rabbit MDR1 antibody (ab170904, Abcam), rabbit MRP1 (ab180960, Abcam), or rabbit CyclinD1 antibody (ab134175, Abcam, USA), then hybridized with a secondary goat anti-rabbit antibody (A0208, Beyotime, China) by turns. The membrane was developed using BeyoECL Plus reagent (Beyotime), and images were collected. Band intensities were quantified using Image J (National Institutes of Health, USA) and normalized to GAPDH.

### Luciferase reporter assay

The TOPflash (wild-type TCF binding sites)/FOPflash (mutant TCF binding sites) luciferase reporter plasmid system (Biovector NTCC, Ltd., Beijing, China) enabled Wnt signaling quantitation in cells transfected with these constructs. For reporter assays, cells were transiently co-transfected with luciferase plasmid and PVT1 silencer plasmid using Lipofectamine 3000. Reporter assays were performed 48 h post-transfection using the dual-lucy assay kit (Vigorous Biotech, Beijing, China), with firefly luciferase used as the baseline and renilla luciferase as the internal control.

### Statistical analysis

All data were shown as means ± SD of five independent experiments and analyzed with GraphPad Prism 5.0 (Graphpad Software, La Jolla, CA). Datasets were compared using Student's *t*-test and one-way ANOVA. *P*<0.05 represented a significant difference.
